# Robust and automatic motion-capture data recovery using soft skeleton constraints and model averaging

**DOI:** 10.1371/journal.pone.0199744

**Published:** 2018-07-10

**Authors:** Mickaël Tits, Joëlle Tilmanne, Thierry Dutoit

**Affiliations:** Numediart Institute, University of Mons, Mons, Belgium; University of Innsbruck, AUSTRIA

## Abstract

Motion capture allows accurate recording of human motion, with applications in many fields, including entertainment, medicine, sports science and human computer interaction. A common difficulty with this technology is the occurrence of missing data, due to occlusions, or recording conditions. Various models have been proposed to estimate missing data. Some are based on interpolation, low-rank properties or inter-correlations. Others involve dataset matching or skeleton constraints. While the latter have the advantage of promoting a realistic motion estimation, they require prior knowledge of skeleton constraints, or the availability of a prerecorded dataset. In this article, we propose a probabilistic averaging method of several recovery models (referred to as *Probabilistic Model Averaging* (PMA) in this paper), based on the likelihoods of the distances between body points. This method has the advantage of being automatic, while allowing an efficient gap data recovery. To support and validate the proposed method, we use a set of four individual recovery models, based on linear/nonlinear regression in local coordinate systems. Finally, we propose two heuristic algorithms to enforce skeleton constraints in the reconstructed motion, which can be used on any individual recovery model. For validation purposes, random gaps were introduced into motion-capture sequences, and the effects of factors such as the number of simultaneous gaps, gap length and sequence duration were analyzed. Results show that the proposed probabilistic averaging method yields better recovery than (i) each of the four individual models and (ii) two recent state-of-the-art models, regardless of gap length, sequence duration and number of simultaneous gaps. Moreover, both of our heuristic skeleton-constraint algorithms significantly improve the recovery for 7 out of 8 tested motion-capture sequences (*p* < 0.05), for 10 simultaneous gaps of 5 seconds. The code is available for free download at: https://github.com/numediart/MocapRecovery.

## Introduction

Motion capture (MoCap) is the process of recording motion data through any type of sensor. Because of the recent evolution of the digital world, motion capture techniques are increasingly used for clinical purposes, sports science, as well as many applications in gaming, animation, and human computer interaction [1–6]. State-of-the-art MoCap systems rely on optical technologies, either marker-based (e.g. Vicon [7]), or markerless (e.g. Microsoft Kinect [8]). The former allow accurate tracking of optical markers (generally consisting of retro-reflective spheres) fixed on a suit. The latter use computer vision techniques like background suppression, silhouette extraction and skeleton reconstruction in RGB images or depth maps [9–11]. Besides their advantages and drawbacks, which is beyond the scope of this work, both techniques share an unavoidable issue: missing data. At recording time, if a marker or a body part is hidden from all cameras, its trajectory cannot be completely recorded, resulting in a gap in the MoCap data. Several issues may cause gaps, including occlusions, marker reflection quality, lighting condition, calibration or the limited area covered by the system. These gaps make it difficult and sometimes impossible to use the data [12–14]. A number of methods have already been proposed to address this issue, based on various techniques. One basic method is direct interpolation. From an incomplete trajectory of a marker, the coordinates over time can be interpolated using standard methods, such as linear, spline or monotone piecewise cubic interpolation [15], amongst others. Those methods are sufficient for small gaps (typically less than 0.5 second for human full-body motion [12]), but are ineffective for larger gaps. More advanced time-series interpolation methods have been proposed, based on linear dynamic systems [16], Gaussian process dynamic models [17], or Kalman filters [13].

Other methods are based on the fact that MoCap data generally consist of highly related trajectories of several markers, due to fixed bone length and to limited degrees of freedom in the skeleton. Expressing the incomplete trajectory using local coordinates, based on trajectories of three additional markers or based on a rigid body position and orientation, can be used to improve recovery [14]. Such coordinate transformation should reduce the variance of the trajectory representation, thereby easing the interpolation process. However, the three markers used for coordinate system transformation must have similar trajectories to the incomplete marker for the process to be efficient. This method thus highly depends on the number of complete marker trajectories available in the data.

Yet other methods for recovering missing data are based on human motion modeling, trained on a pre-recorded dataset. Liu and McMillan [12] trained a global linear model and a set of local linear models from a training set of MoCap data. The local models are defined using segmentation with probabilistic principal component analysis (PCA), and K-means clustering. They first used the global model to recover missing data, then, from the results, they assigned a local model to each frame using a Random Forest classifier. On the other hand, Chai and Hodgins [18] directly retrieved nearest neighbors of incomplete frames in a dataset, and trained a local linear model from these neighbors to recover the missing data. These methods are not fully automatic as they need a large dataset for the training of the models. Moreover, the data to recover must have the same marker disposition as the one used in these pre-trained models. It means that for a new type of MoCap data (with a different marker disposition), an entire dataset must be recorded to train a new model.

Finally, some methods are based on matrix transformation techniques, using PCA [19, 20], singular value thresholding (SVT) [21] or nonnegative matrix factorization (NMF) [22]. These methods consider the entire motion as a matrix, with columns representing 3D components of all marker trajectories, and allow the use of information based on linear relations between the columns to reconstruct a gap in the matrix. The transformations are all based on low-rank properties of MoCap data. A key point with these methods is that a low-rank model of motion is trained on the available data of the motion sequence itself, and does not require a training dataset. These methods are thus automatic and can be used on any MoCap data [23].

A drawback of all previous methods is that the recovered trajectories may not respect human body properties, including bones’ fixed lengths. Motion animations may thus lead to unrealistic results. Yet, other methods are directly based on the body skeleton, forcing marker positions to respect these properties. These constraints were successfully applied to several previously mentioned methods. Li et al. [24] proposed the BoLeRO algorithm, combining skeleton constraints with linear dynamic systems. Tan et al. [25] proposed a skeleton constrained SVT algorithm. Peng et al. [22] adapted NMF to a hierarchical block-based skeleton structure model. However, such methods are generally significantly more computationally intensive as they are based on iterative optimization procedures. Moreover, they are often not automatic, as they are defined for a specific skeleton model based on a pre-defined marker set. Nonetheless, automatic procedures exist to estimate a skeleton structure in MoCap data [26, 27].

Each one of the methods mentioned so far has different advantages and drawbacks, possibly making them more or less effective according to different factors, including gap length, number of markers, motion speed and complexity, and total motion sequence duration. For instance, interpolation techniques are inherently independent of the duration of the entire motion sequence and of the number of markers, unlike matrix-based and machine learning based techniques. The latter indeed require training data based on the frames of the sequence without missing markers, to model the relationship between markers. The quality of the model thus depends on the size of the training set, i.e. sequence duration, and the number of markers. On the opposite, machine-learning techniques may be more robust to gap length or motion complexity than interpolation-based methods [12].

To the authors’ knowledge, most previously proposed automatic MoCap data reconstruction methods are based on low-rank or temporal properties of motion, and use matrix operations to model human motion. Few papers focus on the use of machine-learning techniques such as linear and non-linear regression [28, 29] to model the motion of a missing marker. Moreover, no previous work known to the authors proposes the usage of ensemble learning to use likelihoods of different models and construct a more robust global model from the decisions of an ensemble of others [30, 31].

Therefore, the aim of this research is to propose a probabilistic averaging method that can be used with any ensemble of recovery models, and that enforces movement constraints. This method is referred to below as *Probabilistic Model Averaging* (PMA). The averaging process is based on the posterior likelihoods of the distances between the recovered body points and other markers. To validate the method, we used existing recovery models and developed four new regression-based recovery models, which were used as inputs to the proposed probabilistic averaging method.

## Method

**Fig 1:**
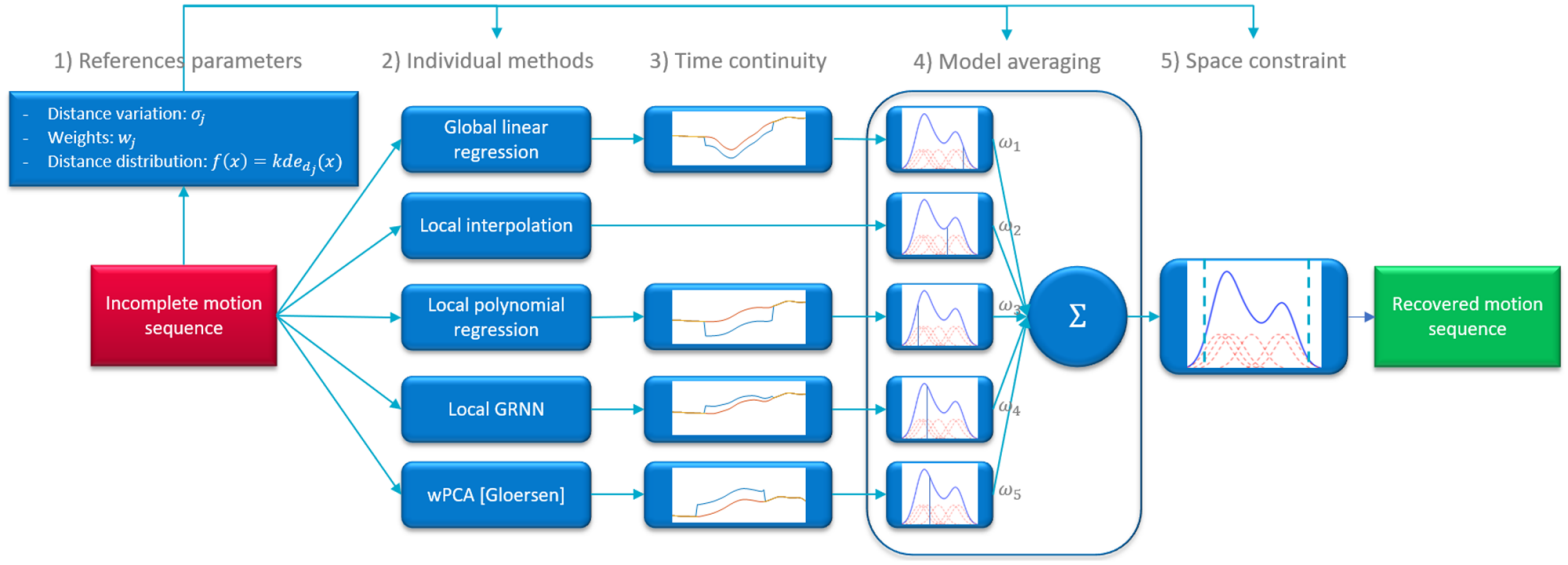
Block diagram of the proposed method. The overall process can be divided in five steps: 1) Extraction of marker trajectories parameters. 2) Individual recovery models. 3) Time constraint: trajectory continuity. 4) Distance-probability weighted averaging. 5) Spacing constraint: reference marker distance likelihoods.

Fig 1 shows the overall approach of our data recovery method, which can be divided into different steps. First, parameters are extracted from each marker trajectory of the motion sequence. These parameters mainly represent relations between markers, and allow identification of related markers (termed as *reference markers* below) and of their distance distributions. Then, various recovery models are applied on the incomplete MoCap sequence, resulting in several candidate recovered sequences. For each candidate, a correction is applied to respect motion continuity (except for interpolation which inherently respects motion continuity). From all resulting individually recovered sequences, a weighted average is applied, in the spirit of ensemble learning systems [30]. Finally, a spacing constraint is applied on the recovered trajectory, enforcing plausibility of the distance with related markers.

Note that it is good practice to center the motion sequence at the outset, by subtracting the mean position of all the markers available in each frame. This process makes it possible to reduce the component of global motion in the sequence, thus reducing motion variations. After the gap recovery process, the mean position is added back to translate the motion sequence to its original trajectories.

In the remainder of the paper, a motion sequence will be considered by a matrix representing all the trajectories of all the recorded markers during the entire sequence. This matrix has the dimension *N* × (3*M*), where *N* is the number of frames of the sequence, and *M* the number of recorded markers. A marker trajectory *p*_*j*_ (*j* ∈ 1, …, *M*) is represented as a matrix *N* × 3.

The method was implemented with Matlab R2017a and the MoCap Toolbox [32]. The code is available for free download at: https://github.com/numediart/MocapRecovery.

### Reference markes definition

All individual recovery models, as well as the proposed spacing constraint, depend on reference markers from which incomplete marker trajectories are to be recovered. We propose an automatic method to define such reference markers, based on inter-marker distance variations, inspired from skeleton-based methods where the intuition is based on the fact that joints of a skeleton have limited distance variations, due to bones’ fixed lengths and limited degrees of freedom. We hypothesize that in many situations an incomplete marker trajectory can be recovered more effectively with methods using reference marker information, due to the close relation between the markers and their references. For instance, the wrist trajectory is more closely related to the elbow or shoulder trajectory than that of the foot. For each marker trajectory to be reconstructed, we can reorder related markers (referred to below as reference markers, or references) using distance standard variation. We denote by *m* the marker trajectory to recover, and *p*_*j*_ any other marker trajectory (*N* − *by* − 3). The distance variation is computed as the standard deviation of the Euclidean distance between *m* and *p*_*j*_:

$$\begin{array}{c} \sigma_{j}=\text{std}(||\left(m-p_{j}\right)\left||\right) \end{array}$$ (1)

The marker *p*_*j*_ with the smallest *σ*_*j*_ is the marker most related to *m*, i.e. the best reference for reconstruction of *m*. All markers can then be sorted as potential references for reconstruction of *m*, according to their distance variation with it.

### Model 1: Global linear regression

An incomplete marker trajectory can be modeled based on trajectories of all present markers as input information. In the present context of human motion data, we have a complex problem involving underlying non-linear relationships, with potentially important quadratic or circular components, due to angular motions of skeleton segments. However, for the method to be fully automatic, the model training data are limited to the incomplete sequence to recover itself, i.e. *N* frames minus the missing frames.

Because many input variables are considered for the design of the model (3 × *M*), the model must be simple, to avoid overfitting. Accordingly, we selected linear regression [28] for that task. Assuming that marker with lower distance variation *σ*_*j*_ are more suited for predicting the missing marker trajectory, we defined a threshold *θ* on *σ*_*j*_, and avoided markers with *σ*_*j*_ > *θ* to simplify the model. This way, only relevant markers (numbered *M*_*X*_) are used to model the position of the incomplete marker. This threshold was experimentally set to 50 mm in this research, as it gave the best results for the dataset, consisting of eight motion sequences with 41 markers (see Results, Table 1). Intuitively, a larger threshold could be considered for data with fewer available markers.

**Table 1:** Motion sequences used in the methods comparison.

File name	Type of motion	#markers	Duration (s)	#frames	fps
HDM_mm_01-02_03_120 (HDM1)	Locomotion on the spot	41	33.25	3990	120
HDM_mm_02-02_02_120 (HDM2)	Shelf (while walking)	41	70.83	8500	120
HDM_mm_03-02_01_120 (HDM3)	Kicking and punching	41	63.93	7671	120
HDM_mm_04-01_02_120 (HDM4)	Chair, table, floor	41	62.79	7535	120
HDM_bd_05-01_01_120 (HDM5)	Clapping and waving	41	57.94	6953	120
85_02 (CMU1)	Break dance (Jumptwist)	41	6.75	810	120
85_12 (CMU2)	Break dance	41	37.5	4499	120
135_02 (CMU3)	Martial art (Empi)	41	43.35	5202	120

Motion files from CMU and HDM05 MoCap databases used to test the methods.

Let *m* be missing between frames *n*_1_ and *n*_2_. In other words, the rows from *n*_1_+ 1 to *n*_2_ − 1 of the matrix *m* must be recovered. A linear regression is performed on each axis *i* of *m*. Let $X={\left[p_{j}\right]}_{j:\sigma_{j}<\theta ,p_{j}\neq m}$ be the design matrix *N* − *by* − (3 × *M*_*X*_), including all marker trajectories *p*_*j*_ with *σ*_*j*_ < *θ*, except the incomplete marker trajectory itself *m* (in practice, we add an intercept column to the design matrix, all missing frames are excluded, and we use only markers always present during the gap of *m* to recover). Denote by *X*(*n*) as the n-th row of *X*, *m*_*i*_ the *i*^*th*^ column (or spatial axis) of *m* (*i* ∈ 1, 2, 3), and *m*_*i*_(*n*) its n-th element. The missing part of *m* is recovered using the following equations:

$$\begin{array}{cc} \beta_{i}= & {\text{argmin}}_{b_{i}}{\left(\sum_{n}{\left(X\left(n\right)\cdot b_{i}-m_{i}\left(n\right)\right)}^{2}\right)}_{i=\left\{1,2,3\right\},n\in \left\{1,...,N\right\}\backslash \left\{n_{1}+1,...,n_{2}-1\right\}} \end{array}$$ (2)

$$\begin{array}{cc} \tilde{m}= & {\left[X\cdot \beta_{i}\right]}_{1\leq i\leq 3} \end{array}$$ (3)

where *β*_*i*_ is the vector of regression coefficients of *m*_*i*_ extracted through least square error minimization, and $\tilde{m}$ is the trajectory recovered with global linear regression. In practice, computation of $\tilde{m}$ is needed only for the missing frames.

### Model 2: Local interpolation

To simplify the modeling of the incomplete marker trajectory, skeletal motion properties involving strong relations between markers can be considered. A local coordinate system can be defined based on three references, and hence reduce the variance of the trajectory representation [14].

Our second algorithm performs a local interpolation, i.e. an interpolation performed in a local reference defined by three other markers (the references).

Denote by *p*_1_, *p*_2_, and *p*_3_ the first three reference markers (ordered by *σ*_*j*_, see Eq 1), used to recover *m*. Define a local coordinate system based on these markers, with three orthonormal vectors (*u*_1_,*u*_2_, *u*_3_) at each time (or frame) *n* (here *n* indicates the row of a matrix (1 ≤ *n* ≤ *N*) For instance, *p*_2_(*n*) is the *n*^*th*^ row of *p*_2_, i.e. a 1 × 3 vector):

$$\begin{array}{ccccccc} & & v_{1}\left(n\right)= & p_{2}\left(n\right)-p_{1}\left(n\right),\; & u_{1}\left(n\right)= & \frac{v_{1}\left(n\right)}{\left|\right|v_{1}\left(n\right)\left|\right|} & \end{array}$$ (4)

$$\begin{array}{ccccccc} & & v_{2}\left(n\right)= & v_{1}\left(n\right)\times \left(p_{3}\left(n\right)-p_{1}\left(n\right)\right),\; & u_{2}\left(n\right)= & \frac{v_{2}\left(n\right)}{\left|\right|v_{2}\left(n\right)\left|\right|} & \end{array}$$ (5)

$$\begin{array}{ccccccc} & & & & u_{3}\left(n\right)= & u_{1}\left(n\right)\times u_{2}\left(n\right) & \end{array}$$ (6)

where × indicates the cross product. *m* can then be projected into the local coordinate system:

$$\begin{array}{cc} P= & \left[u_{1}{\left(n\right)}^{T}\;u_{2}{\left(n\right)}^{T}\;u_{3}{\left(n\right)}^{T}\right] \end{array}$$ (7)

$$\begin{array}{cc} m_{l}\left(n\right)= & (m\left(n\right)-p_{1}\left(n\right))\cdot P \end{array}$$ (8)

*P* is the projection matrix, and *m*_*l*_ the (projected) local trajectory.

*m*_*l*_ can be interpolated with simple linear interpolation. The recovered local trajectory can then be projected back into the original coordinate system:

$$\begin{array}{cc} {\tilde{m}}_{l}\left(n\right)= & \left\{ \begin{array}{c} m_{l}\left(n_{1}\right)+\frac{n-n_{1}}{n_{2}-n_{1}}\cdot \left(m_{l}\left(n_{2}\right)-m_{l}\left(n_{1}\right)\right),for\;n=\left\{n_{1}+1,...,n_{2}-1\right\}\\ m_{l}\left(n\right),\;\text{otherwise} \end{array}\right. \end{array}$$ (9)

$$\begin{array}{cc} \tilde{m}\left(n\right)= & {\tilde{m}}_{l}\left(n\right)\cdot P^{-1}+p_{1}\left(n\right) \end{array}$$ (10)

The interpolation is possible under the condition that all three references are present at frames *n*_1_ and *n*_2_. Also, if there are missing frames in a reference during the gap (*n* ∈ {*n*_1_+ 1, …*n*_2_ − 1}), the incomplete marker trajectory *m* will only be partially recovered. In this case, we can iterate the process with other references on the residual gap (i.e. *p*_1_, *p*_2_, *p*_4_ if *p*_3_ was missing during the gap of *m* to fill, and so on) until *m* is completely recovered.

### Model 3: Local polynomial regression

As just discussed, local interpolation takes advantage of markers relations by performing an interpolation in a local coordinate system. To further use that advantage, we can model and predict the position of the missing marker from its neighborhood (the local reference markers), using regression. As the number of input variables is much lower than for global regression, we can use a more complex model, able to model the non-linear relations between marker trajectories.

Our third algorithm is based on polynomial regression in the local coordinate system. First, the trajectory to recover is projected into a local coordinate system (*m*_*l*_) defined by reference markers *p*_1_, *p*_2_, and *p*_3_ (see Eq 8). For each local coordinate of the marker to recover, a polynomial regression is performed, using reference markers local coordinates as input variables. In practice, only three input local coordinates are useful: the origin of the system is located in *p*_1_ (*p*_1_ = (0, 0, 0)), the new x-axis passes through *p*_2_, giving $p_{2}=\left(x_{p_{2}},0,0\right)$, and the new y-axis is normal to the plane passing through *p*_1_, *p*_2_, and *p*_3_, giving $p_{3}=\left(x_{p_{3}},0,z_{p_{3}}\right)$. Finally, the input set is composed of three variables:

$$\begin{array}{c} X^{l}=\left\{x_{p_{2}},x_{p_{3}},z_{p_{3}}\right\} \end{array}$$ (11)

For polynomial regression, the input variable set *X*^*l*^ is extended to quadratic polynomials in the input variables, leading to a set of 9 variables:

$$\begin{array}{c} X^{q}=\left\{X_{1}^{l},X_{2}^{l},X_{3}^{l},{X_{1}^{l}}^{2},{X_{2}^{l}}^{2},{X_{3}^{l}}^{2},X_{1}^{l}\cdot X_{2}^{l},X_{1}^{l}\cdot X_{3}^{l},X_{2}^{l}\cdot X_{3}^{l}\right\} \end{array}$$ (12)

The regression model is trained on the frames of the motion sequence where all markers (*m*_*l*_, *p*_1_, *p*_2_, *p*_3_) are present. The trained model is then used to predict all missing values of *m*_*l*_:

$$\begin{array}{cc} \beta_{i}= & {\text{argmin}}_{\beta_{i}}{\left(\sum_{n}{\left(X^{q}\left(n\right)\cdot \beta_{i}-m_{i}\left(n\right)\right)}^{2}\right)}_{i=\left\{1,2,3\right\},n\in \left\{1,...,N\right\}\backslash \left\{n_{1}+1,...,n_{2}-1\right\}} \end{array}$$ (13)

$$\begin{array}{cc} {\tilde{m}}_{l}= & {\left[X^{q}\cdot \beta_{i}\right]}_{1\leq i\leq 3} \end{array}$$ (14)

Finally, the recovered local trajectory ${\tilde{m}}_{l}$ can be projected back into the original coordinate system (see Eq 10). Like local interpolation, this method is processed iteratively on sorted references until the trajectory of $\tilde{m}$ is completely recovered.

### Model 4: Local generalized regression neural network

Generalized Regression Neural Network (GRNN) is a non-linear regression method, already used in various applications [33]. It is a variant of an artificial neural network, consisting of four layers: the input layer, a radial basis layer, a summation layer and the output layer. GRNN allows to estimate any arbitrarily complex function, given a sufficient number of observations (generating the radial basis kernels). Comparatively to standard neural networks, GRNN does not require an iterative training. Moreover, as the output of the model is bounded by the extrema of the training dataset, a GRNN can only give physically meaningful outputs [34]. It means for instance that a GRNN should not estimate marker positions with highly implausible distances.

The proposed algorithm applies a GRNN on local variables *X*^*l*^, to model and predict the local trajectory *m*_*l*_. The GRNN is thus trained with three input variables (in practice, each input variable is standardized by subtracting its mean and dividing it by its standard deviation). (*X*^*l*^, see Eq 11) and three output variables (${\tilde{m}}_{l}$), according to:

$$\begin{array}{c} {\tilde{m}}_{l}\left(n\right)=\frac{\sum_{k}m_{l}\left(k\right)\cdot \exp{\left(-\frac{\left|\right|X^{l}\left(n\right)-X^{l}\left(k\right){\left|\right|}^{2}}{2s^{2}}\right)}_{k=\left\{1,...,N\right\}\backslash \left\{n_{1}+1,...,n_{2}-1\right\}}}{\sum_{k}\exp{\left(-\frac{\left|\right|X^{l}\left(n\right)-X^{l}\left(k\right){\left|\right|}^{2}}{2s^{2}}\right)}_{k=\left\{1,...,N\right\}\backslash \left\{n_{1}+1,...,n_{2}-1\right\}}} \end{array}$$ (15)

Parameter *s* determines the smoothness of the regression, and was experimentally set to 0.3 in this research (for standardized input variables), as it gave the best results for the dataset tested. Intuitively, a larger *s* could be chosen to recover slow motions, and a smaller one for sharp and fast motions.

Like the other local recovery models, local GRNN process is iterated on sorted marker references until the trajectory of $\tilde{m}$ is completely recovered.

All these individual models can be used independently to recover a trajectory, leading to several candidates. We explain in the next sections how these candidates are further processed and combined to produce a more robust recovery.

### Time constraint: Trajectory continuity

Human motion time series are limited by two major constraints:

- a spacing constraint, defined by limited ranges of motion and fixed bone lengths;
- trajectory continuity, due to body inertia.

All recovery techniques that are based on interpolation intrinsically respect the continuity constraint. However, this is not the case of predictive models. To enforce continuity on recovered data, we can add a linear correction ramp:

$$\begin{array}{cc} \delta_{n_{1}}= & \tilde{m}\left(n_{1}\right)-m\left(n_{1}\right) \end{array}$$ (16)

$$\begin{array}{cc} \delta_{n_{2}}= & \tilde{m}\left(n_{2}\right)-m\left(n_{2}\right) \end{array}$$ (17)

$$\begin{array}{cc} \delta (n)= & \left\{ \begin{array}{c} \delta_{n_{1}}+\frac{n-n_{1}}{n_{2}-n_{1}}\cdot \left(\delta_{n_{2}}-\delta_{n_{1}}\right),for\;n=\left\{n_{1}+1,...,n_{2}-1\right\}\\ \left(0\;0\;0\right),\;\text{otherwise} \end{array}\right. \end{array}$$ (18)

$$\begin{array}{cc} \overset{˘}{m}= & \tilde{m}-\delta \end{array}$$ (19)

**Fig 2:**
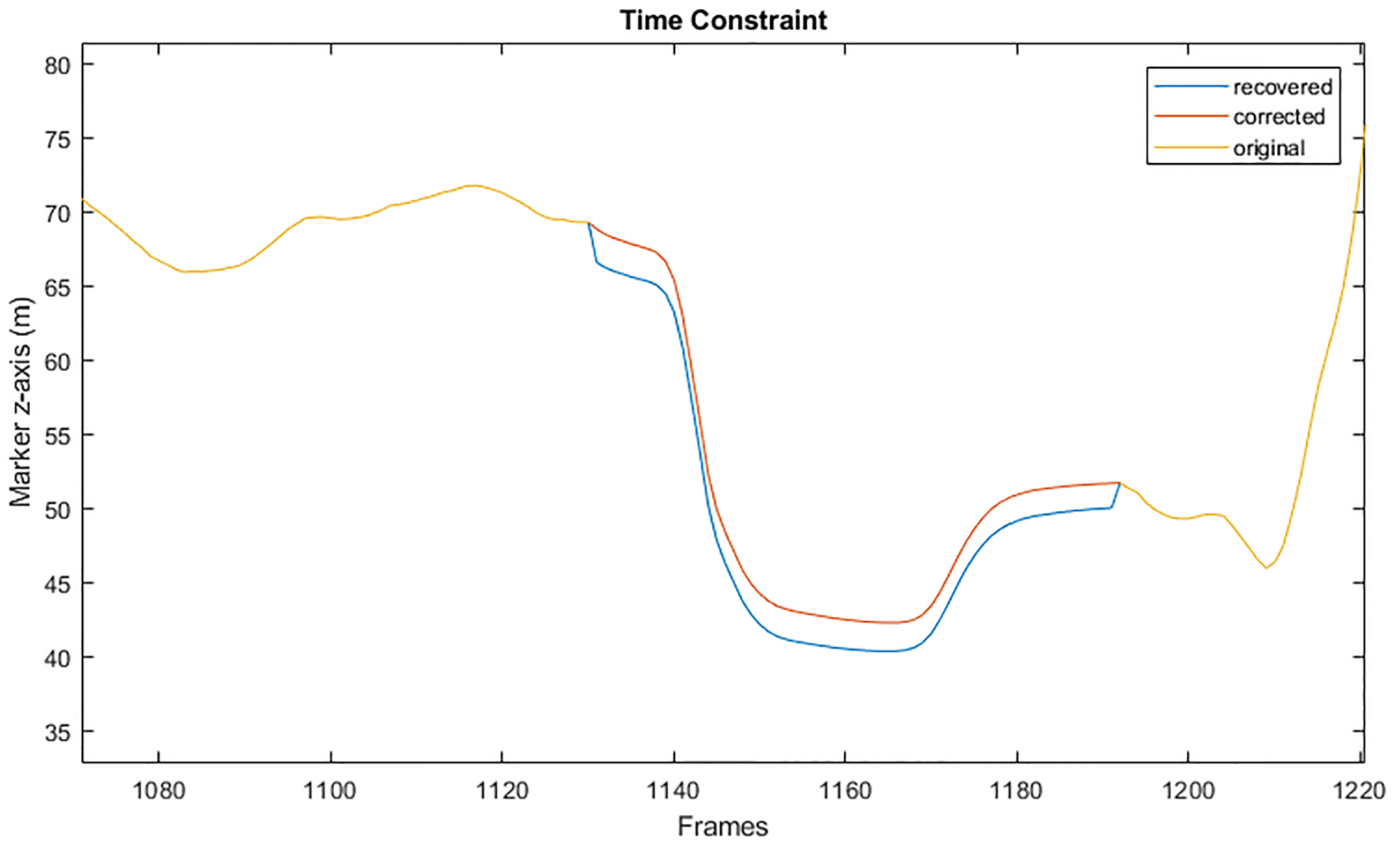
Trajectory continuity correction. The yellow curve shows incomplete data of a marker trajectory (*m*) on which a gap was introduced between frames 1130 and 1190 (only *z*-axis is shown). The blue curve represents the recovered data ($\tilde{m}$), and the red curve shows the corrected data using trajectory continuity constraint ($\overset{˘}{m}$) (see Eqs 16–19).

For each axis, we compute the difference between the real value and the predicted value at each border ($\delta_{n_{1}}$ and $\delta_{n_{2}}$), and subtract from the recovered trajectory a linear ramp from $\delta_{n_{1}}$ to $\delta_{n_{2}}$. An example of this correction is illustrated in Fig 2.

### Probabilistic Model Averaging (PMA)

Depending on the context, each model can be more or less effective, making difficult the choice of the best model, and the development of a robust recovery method. To address this issue, we propose a model averaging method, based on the posterior likelihoods of the distances between the recovered body points and other markers. This method is inspired from Bayesian model averaging [31].

We estimate the *a posteriori* probability of each predicted location according to their distance to reference markers. For references *p*_1_, *p*_2_, and *p*_3_, we estimate the distance distribution with *m* throughout the entire motion sequence on non-missing frames, using kernel smoothing density estimation (“kde” [35], used with Silverman’s rule of thumb to choose the bandwidth of the kernel estimator [36]). For each recovery method *k*, a weight is computed:

$$\begin{array}{cc} d_{j}= & \left|\right|p_{j}-m\left|\right| \end{array}$$ (20)

$$\begin{array}{cc} f_{j}\left(x\right)= & {\text{kde}}_{d_{j}}\left(x\right) \end{array}$$ (21)

$$\begin{array}{cc} {\overset{˘}{d}}_{jk}\left(n\right)= & \left|\right|p_{j}\left(n\right)-{\overset{˘}{m}}_{k}\left(n\right)\left|\right| \end{array}$$ (22)

$$\begin{array}{cc} \omega_{k}\left(n\right)= & f_{1}\left({\overset{˘}{d}}_{1k}\left(n\right)\right)\cdot f_{2}\left({\overset{˘}{d}}_{2k}\left(n\right)\right)\cdot f_{3}\left({\overset{˘}{d}}_{3k}\left(n\right)\right) \end{array}$$ (23)

Here *ω*_*k*_ is the weight of the trajectory ${\overset{˘}{m}}_{k}$ recovered with method *k*, and *f*_*j*_ is the estimated probability density function of the distance between *m* and the reference marker *p*_*j*_.

We then compute a weighted average of all the recovered trajectories:

$$\begin{array}{c} \bar{m}\left(n\right)=\frac{\sum_{k=1}^{K}{\overset{˘}{m}}_{k}\left(n\right)\cdot \omega_{k}\left(n\right)}{\sum_{k=1}^{K}\omega_{k}\left(n\right)} \end{array}$$ (24)

*K* is the number of individual models used for the recovery.

This process allows to give more importance to most likely recovered trajectories, according to their distance with other markers. In the remainder of the paper, we denote this method as *Probabilistic Model Averaging* (PMA).

### Spacing constraint: Reference marker distance confidence interval

A final step is applied on the recovered trajectory. Knowing the probability density distribution of the distance ||*p*_1_ − *m*||, i.e. *f*_1_ (see Eq 21), we can check if the distance of the recovered trajectory with *p*_1_ respects the confidence interval:

$$\begin{array}{c} CI=\{x:F_{1}\left(x\right)\in \left[0.05;0.95\right]\}, \end{array}$$ (25)

where *F*_1_ is the cumulated probability density function estimation of the distance ||*p*_1_ − *m*||, i.e. $F_{1}\left(x\right)=\int_{-\infty }^{x}f_{1}\left(\xi \right)d\xi$. The limits of this interval correspond to two spheres centered on *p*_1_, with radii corresponding to:

$$\begin{array}{cc} r_{1}= & \text{arg}\underset{x}{(}F_{1}\left(x\right)=0.05) \end{array}$$ (26)

$$\begin{array}{cc} R_{1}= & \text{arg}\underset{x}{(}F_{1}\left(x\right)=0.95) \end{array}$$ (27)

**Fig 3:**
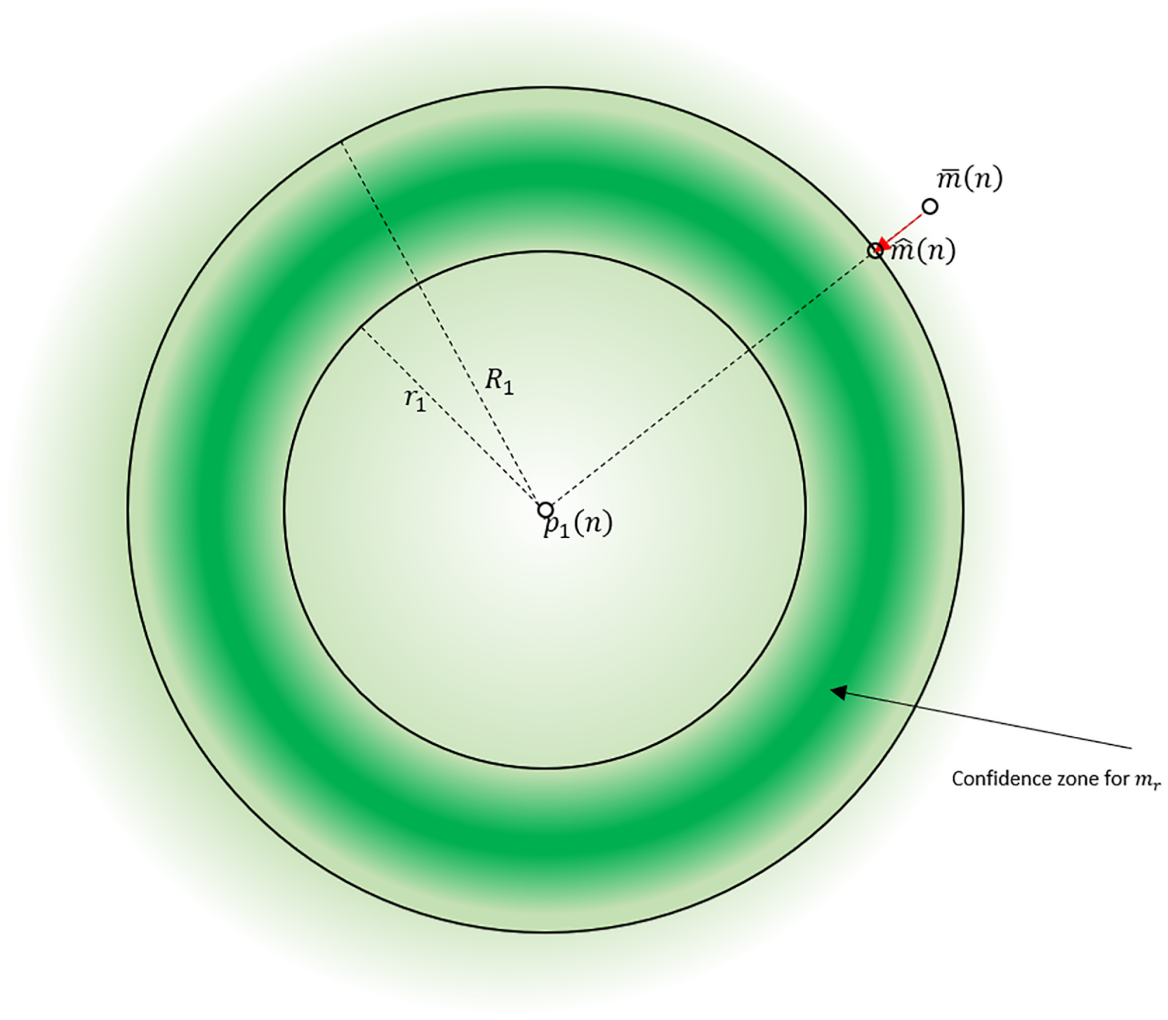
Reference distance soft constraints. The green intensity colormap indicates the probability of presence for the recovered frame. If the recovered frame $\bar{m}\left(n\right)$ is outside the confidence zone (delimited by spheres of radii *r*_1_ and *R*_1_), it is projected onto the closest point in this confidence zone ($\hat{m}\left(n\right)$).

If the recovered trajectory is outside these limits, it is projected onto the closest limit sphere. Fig 3 illustrates the projection of $\bar{m}\left(n\right)$ onto the limits of the confidence interval. In this example, the recovered frame $\bar{m}\left(n\right)$ is outside the confidence zone, as $x=\left|\right|p_{1}\left(n\right)-\bar{m}\left(n\right)\left|\right|>R_{1}$. The red arrow shows $\hat{m}\left(n\right)$, projection of the recovered frame $\bar{m}\left(n\right)$ onto the *R*_1_-radius sphere centered at *p*_1_, to fit the soft skeleton constraint. If the recovered frame is already in the confidence zone, no correction is applied: $\hat{m}\left(n\right)=\bar{m}\left(n\right)$. This process is thus used only if the recovered frame has low a posteriori probability, i.e. an unusual distance with its first reference marker *p*_1_.

A stricter version of the spacing constraint can be applied by recursively projecting the recovered point onto the CI obtained for several reference markers (e.g. the first three references *p*_1_, *p*_2_ and *p*_3_), iteratively until the point is at a plausible distance of each reference.

### Experiments

To validate our method, we tested each method used individually as well as the method combination with PMA. For such testing, we used the online CMU MoCap database (http://mocap.cs.cmu.edu/) [37] and the HDM05 database (http://resources.mpi-inf.mpg.de/HDM05/) [38]. They contain a high number of various motion sequences, and they have been used by much of the related work [12, 20, 22, 24, 25, 39]. Table 1 shows the motion sequences selected for the methods comparison. Motion sequences were selected to include a large variety of motions, in terms of complexity, type of motion, and duration.

The performance of the recovery method on a motion sequence may depend on different factors, including:

- The number of incomplete and complete marker trajectories
- The length of the gaps
- The duration of the sequence
- The complexity or periodicity of the motion

To analyze the performance of each method according to these factors, we introduced three concomitant gaps into our motion sequences, at random locations (uniformly distributed random markers and frames). We applied each method on these incomplete motion sequences, and extracted the recovery error for each method:

$$\begin{array}{c} \epsilon =\frac{1}{g}\sum_{j=1}^{g}\frac{1}{n_{2_{j}}-\left(n_{1_{j}}+1\right)}\sum_{n=n_{1_{j}}}^{n_{2_{j}}}\left|\right|{\hat{m}}_{j}\left(n\right)-m_{j}\left(n\right)\left|\right| \end{array}$$ (28)

*g* is the number of random gaps created, $n_{1_{j}}$ and $n_{2_{j}}$ delimit the location (in frames) of the randomly introduced gap *j*, and *m*_*j*_ and ${\hat{m}}_{j}$ are respectively the original and the recovered trajectories. We iterated this process 20 times with different random gap locations, and a mean recovery error was extracted from all iterations to estimate the general performance of each method. To analyze the influence of the duration of the sequence, fragments with different duration were extracted from each motion file.

Our method performances were compared to related work available online, namely the BoLeRo algorithm from Li et al. [24] (Matlab code available for download at: https://github.com/lileicc/dynammo) and the weighted PCA-based reconstruction method from Gloersen et al. [20] (Matlab code available for download at: https://doi.org/10.1371/journal.pone.0152616). In the sequel, these methods will be identified with the following numbers and acronyms:

1. Global Linear Regression (GLR)
2. Local Interpolation (LI) [14]
3. Local Polynomial Regression (LPR)
4. Local GRNN (LGRNN)
5. weighted PCA-based method (PCA) [20]
6. BoLeRo algorithm with soft bone constraints (BoLeRo) [24]

We used the soft bone constraints version of the BoLeRo algorithm, with 16 hidden dimensions as proposed by Li et al. [24]. The PCA-based method was used with the parameters proposed by their authors, using the consecutive reconstruction strategy for multiple gaps [20].

All results were obtained in MATLAB R2017a on a computer with Intel Core i7-4712HQ 2.3 GHz and 16 GB RAM running Windows 10.

## Results

In this section, we present the results of the recovery on different simulated incomplete motion sequences. We analyze the influence of gap length, motion sequence duration, the number of incomplete marker trajectories, and the influence of the type of motion.

### Gap length

**Fig 4:**
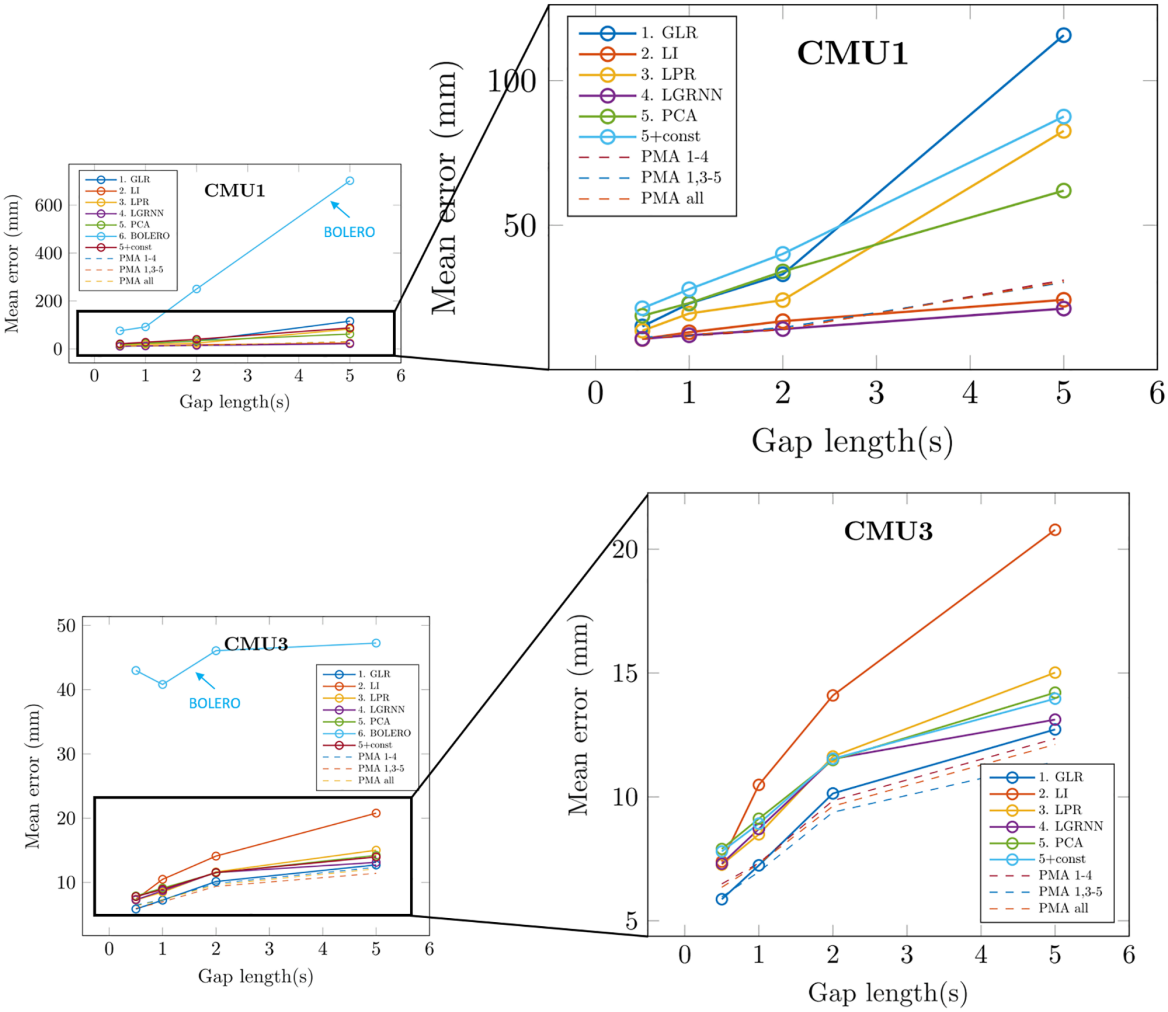
Mean recovery error for different gap sizes and gap recovery methods. Top: CMU1. Bottom: CMU3. Left: results including BoLeRo method. Right: results without BoLeRo method. Each point represents the mean of recovery errors, computed with 20 iterations, of three randomly created gaps of the same length (0.5, 1, 2 or 5 seconds). Solid lines show results for each individual method. Dashed lines show results for distance-probability averages of various combinations of individual methods.

Fig 4 shows the results for two different motion sequences (respectively CMU1 and CMU3). In both cases, BoLeRo gives higher errors than all other methods. Moreover, the processing time, due to the iterative optimization process of the method, is significantly higher than others. For instance, the process duration is above the minute for filling three gaps of 2 seconds in the file CMU3, against less than a second for all other individual methods. For these reasons, and for better graphics readability, BoLeRo is left out in the remaining of the results.

On the right graphs, we can see results for all individual methods, except BoLeRo. Concerning the MoCap sequence CMU1 (top graph), Fig 4 shows a clear separation of each method accuracy, where LGRNN seems to reach the best accuracy (20.2 mm mean error for three random gaps of 5 seconds). Accuracy of all methods seems to decrease with gap size. Concerning the MoCap sequence CMU3 (bottom graph), GLR seems to give the best results, with a mean recovery error of 12.7 mm for three random gaps of 5 seconds.

Fig 4 also shows results of model averaging of several methods (dashed lines). In general, each combination of individual methods (all but 5 (PCA), all but 2 (LI), and all methods) seems to lead to an error comparable to that of the best individual methods in general. Our PMA method thus seems robust to gap size.

### Motion sequence duration

Except for our most basic method based on interpolation (LI), each individual method performance may depend on the motion sequence duration. Indeed, more frames in the sequence means more information (more possible data variation), and more samples for model training.

**Fig 5:**
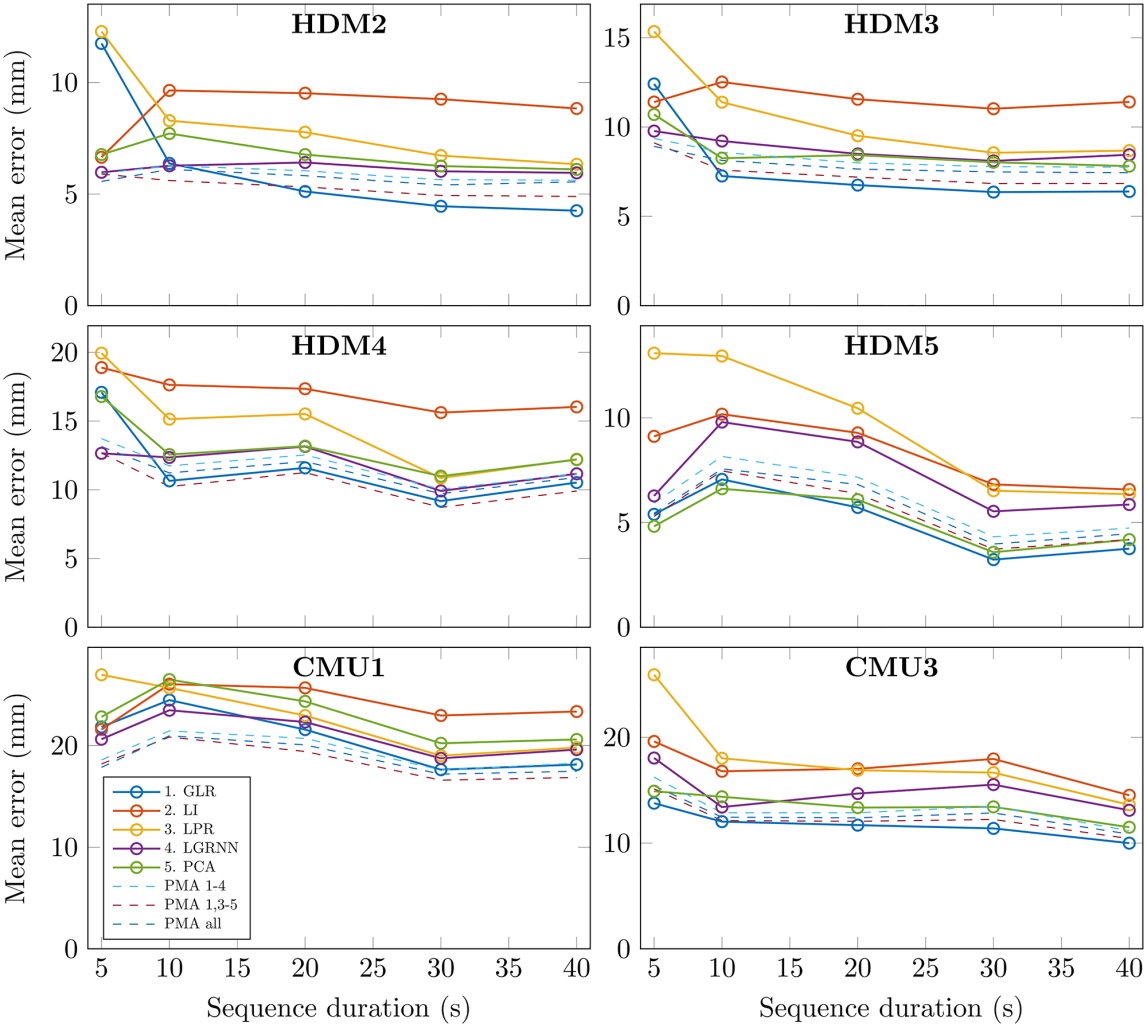
Mean recovery error for different sequence durations and gap recovery methods. To illustrate the influence of sequence duration on performance of gap recovery methods, fragments of different durations were extracted from each motion file. Each point represents the mean of the recovery errors computed on 20 iterations of three randomly created gaps of 1 second. Continuous lines show results for each individual method. Dashed lines show results for PMA with various methods combinations.

To illustrate the influence of sequence duration on performance of gap recovery methods, fragments with different durations were extracted from each motion file. Fig 5 shows the mean recovery error for different sequence durations, for different motion sequences. We can see on each graph that all methods follow similar patterns, showing that their performance highly depends on the specific motion. Nonetheless, for most graphs (except for HDM5), the mean recovery error seems to be higher for sequence durations of 5 seconds, and decreases for a sequence duration of 10 seconds. Beyond that duration, the recovery is not much improved. Concerning individual methods, LGRNN seems to be more robust to sequence duration, compared to other regression methods. For long durations, GLR seems to give the best results of all individual methods.

For all durations and all motion sequences, PMA effectively weights each individual method, hence providing optimal recovery in any context. The best combination is the averaging of all methods but LI (dark red dashed line). Our PMA method is thus robust to motion duration.

### Number of concomitant gaps

**Fig 6:**
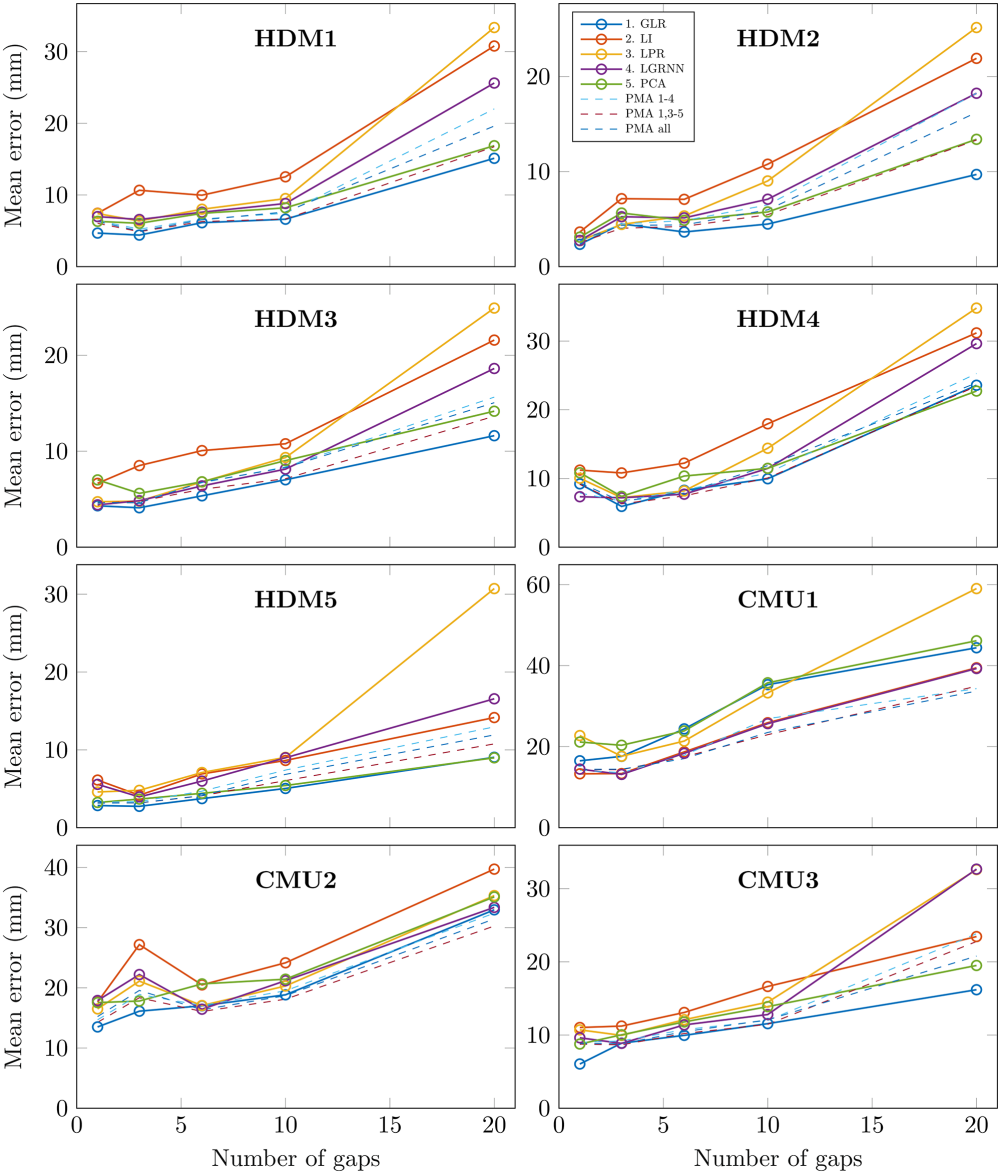
Mean recovery error for different numbers of missing markers and gap recovery methods. Each point represents the mean of recovery errors computed over 20 iterations of a number of randomly created gaps of 1 second (1, 3, 6, 10 or 20 gaps). Solid lines show results for each individual method. Dashed lines show results for distance-probability averages of various methods combinations.

Except for basic interpolation or dynamic filtering methods, the reconstruction quality of one marker trajectory depends on the presence of reference markers. If several markers are missing at the same time during the motion sequence, less information is available for reconstruction. According to the method, the quality of the reconstruction may be influenced differently. Fig 6 shows the mean recovery error of individual methods and their PMA combinations for different motion sequences, and for different numbers of markers missing at the same time (gaps of one second). We can see in general that for all motion sequences and for all methods, the recovery error grows with the number of concomitant gaps. Again, PMA generally give the best results.

### Constraints effect

For all previous results, time and spacing constraints were applied for all individual methods and model averages. To verify the effectiveness of these constraints, PMA reconstruction was tested with and without constraints for each motion sequence, with 200 iterations of three gaps of one second. For each motion sequence, a paired t-test was performed on the mean recovery error of the 200 iterations with and without constraint, as shown in Table 2.

**Table 2:** Effect of constraints on mean recovery error (t-test, n = 200; conditions: 3 gaps of 1 seconds).

Sequence	$\bar{\epsilon }$, no constraint (mm)	$\bar{\epsilon }$, constraint (mm)	Difference (mm)	t-test p-value
HDM1	8.1	6.6	1.5	*p* < 10*e* − 5
HDM2	4.8	4.4	0.4	*p* = 0.46
HDM3	5.5	5.2	0.3	*p* = 0.07
HDM4	8.5	7.4	1.2	*p* = 0.08
HDM5	4.5	3.0	1.5	*p* = 0.001
CMU1	17.1	15.7	1.5	*p* = 0.008
CMU2	14.4	13.5	1.0	*p* < 10*e* − 3
CMU3	10.5	8.4	2.1	*p* < 10*e* − 10

Paired t-test (n = 200) on constraints effect on PMA for the reconstruction of 3 gaps of 1 second, introduced into different motion sequences. Individual methods 1 to 4 were used in this test.

Results show that for almost all tested motion sequences, PMA yields a significant improvement of the recovery method. The constraints did not improve the recovery for the sequence HDM2 (larger p-value), but this may be due to the fact that the recovery error is already low without constraint ($\bar{\epsilon }=4.8mm$).

Table 3 shows a similar analysis in a situation of low marker presence. In this case, 10 simultaneous gaps of 5 seconds were introduced into each motion sequence. We can see that in such situation, PMA’s mean recovery error is much higher, and constraints always improve it significantly, up to 40mm for CMU1.

**Table 3:** Effect of constraints on the mean recovery error (t-test, n = 200; conditions: 10 gaps of 5 seconds).

Sequence	$\bar{\epsilon }$, no constraint (mm)	$\bar{\epsilon }$, constraint (mm)	Difference (mm)	t-test p-value
HDM1	40.9	13.5	27.4	*p* < 10*e* − 10
HDM2	14.4	8.4	6.0	*p* < 10*e* − 3
HDM3	32.2	10.8	21.4	*p* < 10*e* − 10
HDM4	29.8	16.1	13.7	*p* < 10*e* − 8
HDM5	25.0	8.6	16.4	*p* < 10*e* − 10
CMU1	80.8	39.1	41.7	*p* < 10*e* − 10
CMU2	27.7	26.2	1.5	*p* = 0.16
CMU3	22.1	19.6	2.6	*p* = 0.02

Paired t-test (n = 200) on constraints effect on PMA for the reconstruction of 10 simultaneous gaps of 5 seconds, introduced into different motion sequences. Individual methods 1 to 4 were used in this test.

### Synthesis—Mean results

**Fig 7:**
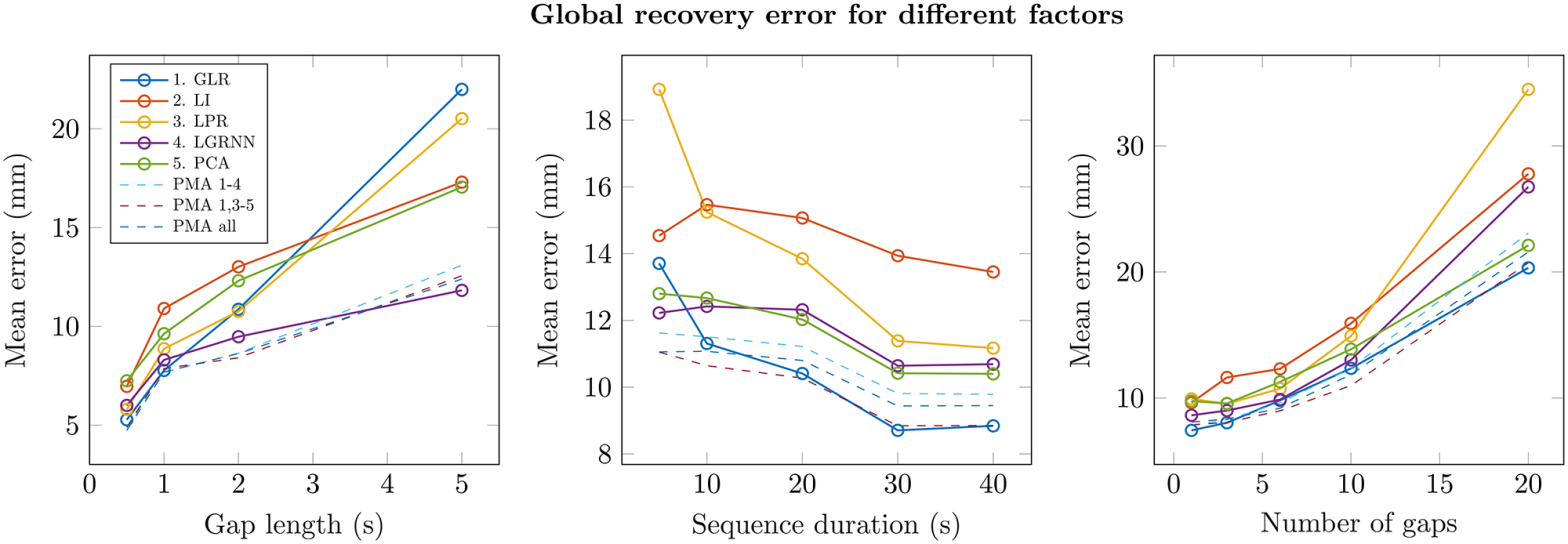
Mean recovery error for different recovery methods, for all test motion sequences. Left: different gap lengths (3 concomitant gaps, total sequence duration); Center: different motion durations (3 concomitant gaps of 1 second); Right: different numbers of concomitant gaps (gaps of 1 second, total sequence duration). Each point represents the mean of recovery errors computed over 20 iterations of a number of randomly created gaps. Solid lines show results for each individual method. Dashed lines show results for PMA with various individual methods combinations.

As a synthesis, Fig 7 shows the mean results of each method, obtained from the mean of the recovery errors on all the selected motion sequences. It can be seen that the various PMA combinations give more robust reconstruction regardless of the type of motion, the gap length (left graph), the duration of the motion sequence (center graph) and the number of incomplete marker trajectories (right graph). Among the individual methods, there is no clear difference of performance according to gap length. The local GRNN method seems more robust to gap length: it allows to recover three concomitant gaps of 5 seconds with a mean error of 10 mm. All other methods lead to a mean error above 15 mm. The local GRNN seems to be more robust to sequence duration. A duration of 5 seconds (with 41 markers, 120 fps) allows to train an effective model to reconstruct three concomitant gaps of 1 second with a mean error of 12 mm. However, for a longer sequence (40 seconds), GLR gives the best results, with a mean error of 9 mm. All individual methods are highly sensitive to the number of concomitant gaps, and thereby to the number of markers available to predict the missing trajectories. Finally, PMA systematically improves the gap recovery, independently of motion type, gap length, sequence duration or number of missing markers. As the local interpolation method seems to be the less effective, the best method combination is the averaging of methods 1, 3, 4 and 5.

## Discussion

Our PMA method presents several advantages compared to the available state of the art. It is fully automatic and does not require any prior knowledge or any pre-trained model. It can be used on MoCap data recorded with any marker set. Graphical results show that PMA is robust to various factors, including gap length, sequence duration, the number of simultaneous gaps, and the type of motion. Additionally, the use of temporal and spacing constraints significantly improves the reconstruction, especially in challenging conditions (see Table 3).

Figs 4–7 show results where spacing and time constraints were applied to each individual method. These constraints may indeed be applied afterwards to any prediction method. The improvement of recovery after method combination is hence exclusively due to PMA, and confirms its effectiveness. In terms of quantitative results, no individual method shows better performance in general. All individual methods are more or less sensitive to the context, including motion type, gap length, sequence duration or the number of simultaneous missing markers. In contrast, PMA seems to take advantage of every individual method, improving the robustness of the recovery algorithm. Moreover, as averaging is based on distance with reference markers, PMA partly takes skeleton constraints into account.

Our methods could not be compared to some recently proposed methods, due to the unavailability of the code. However, the recent skeleton-constrained SVT method from Tan et al. (2015) [25] (not included in our study), based on both skeleton constraints and low rank properties, achieved similar results to BoLeRo [24]: their improvement over BoLeRo mainly lies in execution time, as explained in [25]. Their constraint-fitting optimization method converges significantly faster than BoLeRo.

The effectiveness of the method proposed in the present study, including PMA and time and spacing constraints, is independent of the individual recovery model. It can theoretically be applied to any other set of individual recovery models in the future, possibly leading to better performance.

### Limitations

The methods included in the present study rely on several parameters, including the threshold for reference marker selection in GLR, the smoothing parameter in LGRNN, parameters from w-PCA [20], as well as confidence interval thresholds for spacing constraints. All these parameters were experimentally chosen in this research, as they gave the best results for the dataset tested (see Table 1). The user should adapt these parameters for her/his own data if necessary. Optimal parameters could depend on MoCap data, including for instance the number of recorded markers, their particular placement, the complexity or speed of the motion, data accuracy, or noise due to marker vibrations or camera quality. For instance, a larger threshold for the linear regression model could be considered for MoCap data with fewer available markers, and the optimal smoothness parameter for LGRNN could depend on the smoothness of the motion itself.

For the validation of the proposed methods, gaps were introduced into motion sequences at random locations. It is possible that in some particular cases, a marker can be isolated, without any highly related reference. If this marker is missing, it could lead to a poor recovery. This issue thus depends on the placement of markers It is hence relevant to consider this aspect when defining marker placement, to avoid isolated markers. On the other hand, markers placed too close to each other risk to be occluded simultaneously. A trade-off must thus be considered for their placement.

PMA has some limitations in comparison to a method such as BoLeRo [24]. In case of a blackout, i.e. when all markers disappear at the same time, a method based on a predictive filter such as Kalman filter can reconstruct an entire frame, and then use gradient descent or a similar optimization method to fit skeleton constraints, whereas PMA needs at least three present markers as references to evaluate distance probabilities. However, this is an extreme case, which can generally be avoided with an efficient use of the MoCap system.

### Improvement prospects

Our methods could be improved in various ways. First, human motion is not a stationary process [40]. Each individual model might be made more efficient by giving more importance to motion data that are close to the gap to reconstruct. For instance, for each gap, a local model could be trained on a limited time window centered on that gap. The distance probabilities could also be locally defined on a time window. However, this would limit the number of available data for model training.

Secondly, we could use a more complex constraint fitting method, making use of a dynamic model such as the Kalman filter [41] to ensure trajectory continuity. Additionally, an optimization procedure could be used instead of projection for skeleton constraints fitting whenever the inter-marker distance is outside the confidence interval. However, this could significantly increase execution time.

Finally, an original interest in the distance variation density estimation is the possibility to assess the quality of the reconstruction. It could further be used as an indication to identify and verify the most sensitive parts of the data, and possibly reject them and reprocess them with another configuration or method.

### Processing time consideration

It is interesting to note that in human motion data, adjacent frames are very similar if the frame rate is high enough. In this case, data can be easily subsampled without losing much information for model training. This subsampling can drastically decrease computation time, either for computing reference weights (Eq 1), for individual model training, as well as for kernel smoothing density estimation (Eq 21).

Though it is not the initial goal of the proposed algorithm, each individual method based on regression, as well as their combination with PMA could be adapted for real-time purpose. Each individual model and distance distribution estimation can be trained on previously recorded data, and can be effective after a few seconds of recording. In this case, the time constraint would be limited to information about previous data. A Kalman filter would be appropriate for this task.

## Conclusion

We have proposed an original automatic method, Probabilistic Model Averaging (PMA), for robust reconstruction of missing MoCap data. The robustness of our method relies on two major steps:

1. The weighted combination of several models, based on the posterior likelihoods of inter-marker distances.
2. The application of simple but effective constraints, enforcing trajectory continuity and plausible distance of reconstructed trajectories with related markers.

To support and validate our model-averaging method, several reconstruction methods based on regression and local coordinates were proposed, and were found to compete with state-of-the-art methods. Results show that PMA used with the constraints outperforms individual methods in various conditions, including various gap lengths, motion sequence durations and numbers of simultaneous gaps.

Our method has the advantage of being fully automatic. The algorithm is data-driven, and does not need any prior knowledge or any pre-trained model. Moreover, the model averaging and the proposed constraints are general and can be used with any other individual reconstruction method, leading to possible future improvement.

## Supporting information

S1 Code Matlab code. Matlab script containing a Matlab implementation of the proposed method. Note that the maintained version of the code can be found on the Github repository: https://github.com/numediart/MocapRecovery.
(M) Click here for additional data file.
